# Positive selection drives the evolution of a primate bitter taste receptor gene

**DOI:** 10.1002/ece3.7440

**Published:** 2021-03-23

**Authors:** Xiaoyan Dong, Qiufang Liang, Jiaping Li, Ping Feng

**Affiliations:** ^1^ Key Laboratory of Ecology of Rare and Endangered Species and Environmental Protection (Guangxi Normal University) Ministry of Education Guilin China; ^2^ Guangxi Key Laboratory of Rare and Endangered Animal Ecology Guangxi Normal University Guilin China

**Keywords:** bitter taste receptor gene, primates, selective pressure, T2R1

## Abstract

Bitter taste perception is important in food choice of animals, and it is mediated by bitter taste receptor (T2R) containing three regions: extracellular regions (ECs), transmembrane regions (TMs), and intracellular regions (ICs). It is hypothesized that ECs, TMs, and ICs are under different selective pressures, with ECs being unstable while TMs and ICs being constrained. To test this hypothesis, we examined the selective pressures on one of the bitter taste receptor genes‐*T2R1* and its different areas from 35 primates and found that *T2R1* was under neutral evolution but with some positively selected sites in it. Further analyses suggested that TMs, ICs, and the concatenated transmembrane region TM1237 were under purifying selection; in contrast, extracellular regions, the first and second extracellular loop (EL1, EL2), were subject to positive selection with several positively selected sites in them. Therefore, this study supported the above‐mentioned hypothesis. The reason why EL1 and EL2 of *T2R1* have positively selected sites is probably due to their participation in forming the cap‐like structure involved in ligand binding. Positive selection acts as a driving force of the *T2R1* functional differentiation and confers the ability to discern various bitter substances for primates, which could help them to adapt to the changing environment during the evolutionary course.

## Graphical abstract

Positive selection acts as a driving force of the T2R1 functional differentiation and confers the ability to discern various bitter substances for primate, which could help them to adapt to the changing environment during the evolutionary course.

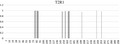

## 1 INTRODUCTION

Taste perception is one of the basic sensation available to animals, and taste system can be used to analyze the food composition and decide whether to eat or reject the food item. Such sense is crucial as animals not only need to intake nutrient substances for survival but also need to avoid toxic compounds (Dutta et al., 2020). Bitter taste, one of the five basic taste qualities (umami, sweet, bitter, sour, and salty), acts as sentinels in defending animals from consuming the potentially toxic and harmful substances which often taste bitter (Chandrashekar et al., 2000; Lu et al., 2017). Bitter taste perception is mediated by the interaction between bitter tastants and bitter taste receptors which are discovered in mouth and throat, and also in extraoral positions such as brain, respiratory tract, lungs, heart, intestines, and bladder (Bloxham et al., 2020; Foster et al., 2013; Garcia‐Esparcia et al., 2013; Jeruzal‐Swiatecka et al., 2020; Shah et al., 2009).

Bitter taste receptors are G protein‐coupled receptors (GPCRs) which are characteristic of 7 transmembrane domains, and they are encoded by *T2R* gene family (Adler et al., 2000; Chandrashekar et al., 2000; Hoon et al., 1999). The evolution of *T2R* genes has been investigated in many aspects, such as the relationship between gene copies and dietary preferences (Li & Zhang, 2014), the ligands for bitter taste receptors (Meyerhof et al., 2010), and the function of specific residuals performing on binding bitter tastants (Tsutsui et al., 2016). Among these studies, some are devoted to revealing the ligands for T2Rs and found that some T2Rs are able to respond to many natural and synthetic compounds; for instance, T2R1 responds to camphor in the owl monkey (Tsutsui et al., 2016) and responds to Amarogentin, Arborescin, Cascarillin, Chloramphenicol, Parthenolide, Picrotoxinin, Thiamine, and Yohimbine in human (Meyerhof et al., 2010); in addition, human T2R1 can also be activated by bitter tasting tri‐ and di‐peptides from all sorts of food proteins (Upadhyaya et al., 2010). T2R38 responds to phenylthiocarbamide (PTC) in human and some other primates (Purba et al., 2017), and so forth.

Adler et al. (2000) reported that the structure of T2Rs can be divided into three functional regions: extracellular regions (ECs), transmembrane regions (TMs), and intracellular regions (ICs). ECs can mediate extracellular ligand binding while TMs are critical to receptor localization and ligand interaction, and ICs participate in interactions with G proteins situated intracellular (Vaidehi et al., 2002; Wooding, 2011). It is hypothesized that selective pressures imposed on ECs, TMs, and ICs are different, with ECs being labile while TMs and ICs restrained (Strotmann et al., 2011), and this hypothesis has been tested in *T2R38* of primates (Wooding, 2011) and *T2R*s of human (Shi et al., 2003). However, whether this hypothesis fits other *T2R* genes or not? How does other *T2R*s evolve in primates? To answer these questions, *T2R1* was selected and examined because its role has been characterized in primates (Tsutsui et al., 2016), and its two‐dimensional structure has been predicted clearly (Adler et al., 2000; Probst et al., 1992), and furthermore, T2R1 is relatively conserved in most species (Hayakawa et al., 2014). Besides, previous research has reported that T2R1 used the binding pocket situated near the extracellular surface to interact with bitter peptides; and the pocket is formed by TM1, 2, 3, 7 domains, with a cap‐like structure formed by EL1 (extracellular loop 1) and EL2 (extracellular loop 2) on it (please refer to Upadhyaya et al. (2010) for detailed structure), and further, ligand binding relied on residues from EL1, 2 and TM1, 2, 3, 7 region (Upadhyaya et al., 2010). In addition, with the fast development of high‐throughput sequencing approaches, the number of fully sequenced genomes are increasing; thus, it is easy to obtain gene sequences information. This provides the great opportunity for investigating the selective pressure of *T2R1* and its different regions. In this study, *T2R1* sequences were collected from the primates with known genomic information, and they would be used to examine the evolutionary process by checking the signature of natural selection and to test the aforementioned hypothesis. The result showed that strong positive selection was present no matter in ECs or in EL1 and EL2, and in the whole *T2R1*gene, many positively selected sites were detected.

## 2 MATERIALS AND METHODS

### 2.1 Genome data

Primate species which have known genomic information were collected and their genomes were downloaded from NCBI (https://www.ncbi.nlm.nih.gov/). The details of these species are included in supplementary materials, Table S1.

### 2.2 Gene identification and collection

*T2R*s are single exon genes with the length of about 900 bp. To identify the *T2R1* gene from the downloaded primate genomes, we referred to the methods applied in previous studies (Feng & Zhao, 2013; Shi & Zhang, 2006), with slight modification. The process are briefly described as follows: first, the published T2R1 protein sequences collected from Li and Zhang (2014), Hayakawa et al. (2014), and Tsutsui et al. (2016) (accession number LC184485, LC184481, LC184483) and T2R1 of human (accession number NM_019599) from GenBank were used to TBLASTN against the downloaded genomes, setting E‐value of 1e‐10 as a cutoff. Second, the blast‐hits shorter than 200 bp were discarded and the sequences which hit on the same scaffold were filtered. Third, the remaining sequences were extended in both 3’ and 5’ directions and extracted from the genomes. Fourth, an alignment between the extracted sequences and their corresponding query sequences were conducted to determine the start codon and stop codon. Fifth, the deduced sequences were searched against GenBank to make sure that they were *T2R*s. Sixth, the resulting sequences were divided into three categories: Sequences with more than 270 codons and a complete open reading frame were viewed as intact genes; sequences with either a start codon or a stop codon were treated as partial genes; sequences with an interrupted reading frame were classified as pseudogenes. Subsequently, intact genes were examined by TMHMM approach (Krogh et al., 2001) to check the existence of seven transmembrane domains. Indeed, some sequences are annotated in the Ensembl database, but according to our previous research (Feng & Zhao, 2013), sometimes the annotations are not correct; thus, we did not consider use the sequences from Ensembl directly.

### 2.3 Sequence analysis and phylogenetic reconstruction

We used mouse *T2R119* gene (accession number NM_020503), which is the 1:1 orthologue to *T2R1*, as an outgroup. Sequences of *T2R1* and mouse *T1R119* were translated into amino acid and then aligned by muscle program (Edgar, 2004); subsequently, the alignment was put into MEGA6 (Tamura et al., 2013) with manual adjustments. Phylogenetic tree of *T2R1* was constructed by using maximum likelihood approach, with 1,000 bootstrap replicates (Felsenstein, 1985), and other parameters were used by default.

### 2.4 Selective pressure analyses

To examine the signatures of natural selection acting on the *T2R1* of primates, we conducted the selective pressure analyses for the obtained *T2R1* genes by using PAML4.7 software (Yang, 2007), with the ML tree of *T2R1* as the guide tree, and referring to its operating manual. We analyzed the relative rate of nonsynonymous substitution (*d*_N_), synonymous substitution (*d*_S_), and *ω*, which is equal to *d*_N_/*d*_S_. When *ω* > 1, the positive selection is prevailed while *ω* < 1 purifying selection is predominant, and in contrast, when *ω* = 1, neutral evolution plays a dominant role. Specifically, we performed the evolutionary analyses by calculating the ratio between rates of synonymous and nonsynonymous substitution, and subsequently, four pairs of model analyses were conducted and compared. Firstly, M0 (one *ω*) and M0 (*ω* = 1) comparison, in M0 (one *ω*) *ω* is allowed to take a single value across a gene but in M0 (*ω* = 1) this value is fixed at 1. Secondly, M1a (nearly neutral) and M2a (positive selection) were compared. M1a assumes two classes of sites, including one fixed with *ω* = 1 and another constrained to *ω* < 1, while M2a is an extension of M1a by adding an additional class of *ω* > 1. Thirdly, M7 (*β*) and M8 (*β* & *ω*) were compared. M7 (*β*) assigns a parameter, *β*, assuming that the *ω* values among sites obey to the *β* probability distribution while constraining *ω* to be smaller than 1, and M8 (*β* & *ω*) assigns an additional class of sites to M7 (*β*), permitting *ω* of some sites to be bigger than 1. Fourthly, M8a (*β* & *ω* = 1), which is similar to M8 but constrains *ω* to be fixed at 1, was compared with M8 (*β* & *ω*) (Yang, 2007). Likelihood ratio tests (LRTs) was used to detect positive selection by comparing twice the log likelihood difference (2dl) with a chi‐square distribution; the degree of freedom can be obtained from the difference of parameter numbers between the two models. The posterior probability (PP) of sites under positive selection (*ω* > 1) was estimated by Bayes Empirical Bayes (BEB) method (Yang et al., 2005), and the sites with a PP > 95% would be selected and recorded. For the comparison of M1a (nearly neutral) versus M2a (positive selection), M7 (*β*) versus M8 (*β* & *ω*), and M8 (*β* & *ω*) versus M8a (*β* & *ω* = 1), after the chi‐square tests, if a significant difference existed between the two models, we concluded that positive selection was prevailed (Zhou et al., 2009).

To examine the selective pressure on different main regions, the regions should be categorized clearly. Location of each region was classified by referring to previous research (Adler et al., 2000; Singh et al., 2011), and the result of an online predicted program TMHMM (http://www.cbs.dtu.dk/services/TMHMM/) (Krogh et al., 2001; Sonnhammer et al., 1998). The external region of T2Rs include one ‐NH_2_ end and three loops, and the internal regions contain one ‐COOH end and three loops (Singh et al., 2011). It is suggested that the second loop (EL2) contains many variable sites and has an abundance of substitution in T2R38 due to its direct contact with tastants (Wooding, 2011), and in T2R1, TM‐1, TM‐2, TM‐3, TM‐7 (TM1237) which form the ligand binding pocket, combines with EL1 and EL2, which form the cap, to interact with ligands; thus, besides EL2, we also test the selective pressure on the concatenated regions TM1237, and EL1 to make clear whether they are under positive selection or not, that is, in this step, not only three major domains: EC, TM, and IC, but also TM1237, EL1, and EL2 were extracted to analyze. In this section, only Model 0 (*ω* = 1) and Model 0 (one *ω*) tests were conducted, and the relevant parameters likelihoods (LnL), *ω*, and *P* values were recorded. When the region was found to be under positive selection, we would further investigate their positively selected sites by comparing three pairs of models: M1a (nearly neutral) versus M2a (positive selection), M7 (*β*) versus M8 (*β* & *ω*), and M8a (*β* & *ω* = 1) versus M8 (*β* & *ω*).

## 3 RESULTS

### 3.1 *T2R1* identification and gene tree analyses

**FIGURE 1:**
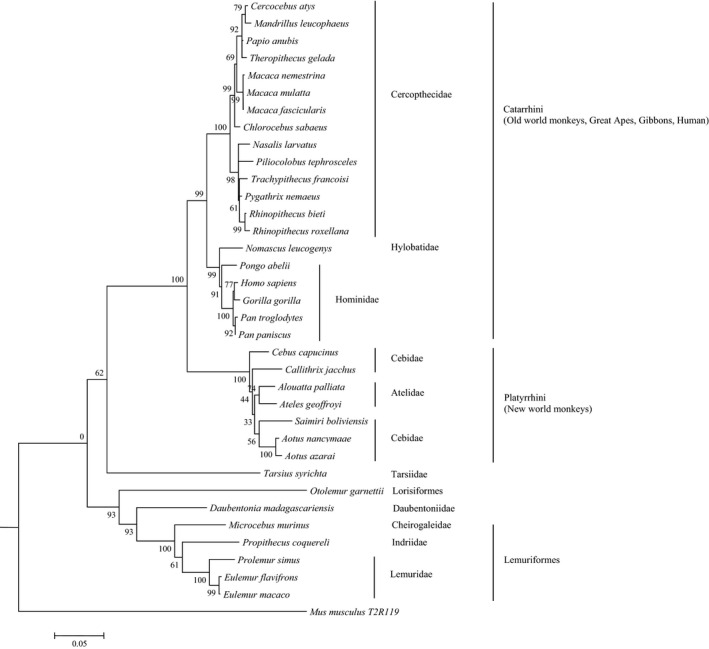
ML tree of *T2R1* gene based on sequences of 35 primate species, and mouse *T2R119* was used as an outgroup

Genomes of 34 primate species (supplementary materials, Table S1) were downloaded and executed the procedure of data mining. Although some species have been searched in other studies for *T2R1*, for example, the *T2R1* of marmoset (*Callithrix jacchus*), gibbon (*Nomascus leucogenys*), rhesus (*Macaca mulatta*), bushbaby (*Otolemur garnettii*), and mouse lemur (*Microcebus murinus*) were annotated by both Li and Zhang (2014) and Hayakawa et al. (2014), they were revised for the current assembly for considering that the current assembly have a higher coverage. As a result, *T2R1* genes of 31 species were identified from the genome data (supplementary materials, Table S2). We failed to find *T2R1* in three species *Colobus angolensis*, *Macaca fuscata*, and *Pongo pygmaeus* probably due to their low genomic quality in the sequence of *T2R1* region. In addition, 3 *T2R1* sequences from *Alouatta palliata*, *Aotus azarai* and *Ateles geoffroyi* respectively were obtained from Tsutsui et al. (2016), and human T2R1 was downloaded from GenBank directly. Thus, in total 35 *T2R1* genes were collected and identified, and they are from 35 species which include 20 Old World monkeys, 7 New World monkeys, 6 species of lemuriformes, and 2 species from tarsiidae and lorisiformes, respectively. The gene tree was reconstructed by Mega6.0 software (Tamura et al., 2013), setting model/method to Kimura 2‐parameter model, and No. of bootstrap replications to 1,000. The resulting ML tree is shown in Figure 1, and the gene tree is largely consistent with previously published phylogenetic tree (Perelman et al., 2011). That is, the lineage of lorisiformes and lemuriformes can be distinguished with the lineage leading to Old World monkeys and New World monkeys, and further, Old World monkeys and New World monkeys form different clades, respectively. In the smaller clade, cercopithecidae, species from cercopithecine and colobine (leaf‐eating monkeys) cluster separately. Within the clades, most relationships accorded with the published phylogenetic tree, but some topologies were different, for example, the cebidae lineage was interrupted by two atelidae species, *Alouatta palliata* and *Ateles geoffroyi*, and in the phylogenetic tree from Perelman et al. (2011), *Cebus capucinus* is closely related to *Saimiri boliviensis*, but in the *T2R1* gene tree here, *Cebus capucinus* is grouped as the sister species of the whole lineage including *Saimiri boliviensis* and *Alouatta palliata*. The incongruence between gene tree and species tree could be due to incomplete lineage sorting (Knowles, 2009; Pollard et al., 2006).

### 3.2 Selective pressure analyses

**FIGURE 2:**
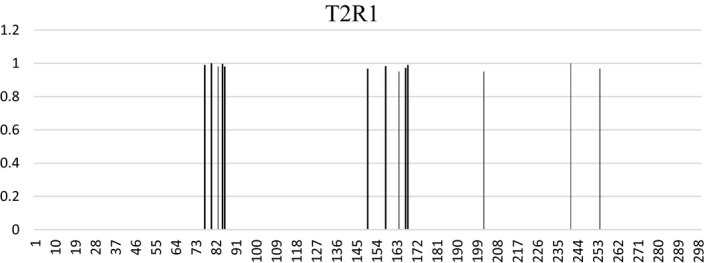
Positively selected sites found in the whole *T2R1* gene sequence. Line with boldface indicted the positively selected sites found in both selective pressure analyses of whole *T2R1* gene sequence and EL1, EL2 alone. In EL1, the position of positively selected sites are 77, 80, 85, and 86, and in EL2, they are 150, 158, 167, and 168

To get the knowledge of selective pressures acting on the primate *T2R1*, several model analyses were conducted by using PAML software (Yang, 2007). The results are shown in Table 1. First, M0 (one *ω*) and M0 (*ω* = 1) analyses were performed and compared by taking *T2R1* as a whole. The result showed that the average *ω* for *T2R1* is 0.94, which was higher than that of other genes in general (Toll‐Riera et al., 2011; Wooding, 2011), indicative of a higher rate of nonsynonymous substitutions relative to the rate of synonymous substitutions in *T2R1*. *P* value for M0 (one *ω*) and M0 (*ω* = 1) comparison is 0.39, bigger than 0.05, indicating that the neutral evolution was not rejected. Second, to identify the potentially positively selected sites in *T2R1*, site model analyses were conducted. Comparison between M1a (nearly neutral) and M2a (positive selection) model revealed that the *P* value for LRT was 4.49E‐16, suggesting that M2a (positive selection) model fitted the data better than M1a (nearly neutral). That was, positive selection was imposed on some sites of *T2R1*. Another two pairs of comparisons M8 (*β* & *ω*) versus M7 (*β*) and M8 (*β* & *ω*) versus M8a (*β* & *ω* = 1) also supported this conclusion, with *P* value of 8.30E‐18 and 3.23E‐17, respectively. Taken together, M2a (positive selection) and M8 (*β* & *ω*) models fitted the data significantly better than their corresponding null models M1a (nearly neutral), M7 (*β*), and M8a (*β* & *ω* = 1). Further, M2a (positive selection) specified 18% of the codons to be positively selected sites (*ω* = 3.01), and M8 (*β* & *ω*) assigned the proportion of positive selection sites (*ω* = 2.82) to 21%, indicating that some sites of *T2R1* had been under positive selection although the whole gene was subject to neutral evolution. In M2a (positive selection) model analysis, 13 positively selected sites (77 M, 80A, 83A, 85L, 86L, 150F, 158K, 164A, 167T, 168L, 202R, 241V, 254I, Figure 2) with PP > 0.95 were detected, and such sites were also found in model M8; meanwhile, 8 additional positively selected sites (9Y, 60L, 148A, 149G, 154Y, 209A, 246L, 250I) were identified in M8. In sum, M2a (positive selection) and M8 (*β* & *ω*) were markedly different from M1a (nearly neutral), M7 (*β*), and M8a (*β* & *ω* = 1), suggesting that the models permitting positive selection fitted the given data significantly better than other models, which was the obvious signal of positive selection.

**TABLE 1:** Likelihood analyses of the branch models and site models in the PAML package for the *T2R1* gene sequences data

Model	Parameter estimates^a^	LnL	2dl (*p* value)	Positively selected sites^a^ (PP > 0.95)
M0 (*ω* = 1)	*ω* = 1	−6268.19		
M0 (one *ω*)	*ω* = 0.94	−6267.82	*M0 (one ω)* versus *M0 (ω* = *1)* 0.74 (0.39)	None
M1a (nearly neutral)	*p_0_* = 0.37, *ω_0_* = 0.10, *p_1_* = 0.63, *ω_1_* = 1.00	−6190.47		
M2a (positive selection)	*p_0_* = 0.32, *ω_0_* = 0.11, *p_1_* = 0.50, *ω_1_* = 1.00, *p_2_* = 0.18, *ω_2_* = 3.01	−6155.13	*M2a* versus *M1a* 70.68 (**4.49E‐16**)	77 M, 80A, 83A, 85L, 86L, 150F, 158K, 164A, 167T, 168L, 202R, 241V, 254I
M7 (*β*)	*p* = 0.19, *q* = 0.09	−6194.11		Not allowed
M8(*β* & *ω* > 1)	*p_0_* = 0.79, *p* = 0.32, *q* = 0.21, *p_1_* = 0.21, *ω_1_* = 2.82	−6154.78	*M8* versus *M7* 78.66 (**8.30E‐18**)	9Y, 60L, **77 M**, **80A**, **83A**, **85L**, **86L**, 148A, 149G, **150F**, 154Y, **158K**, **164A**, **167T**, **168L**, **202R**, 209A, **241V**, 246L, 250I, **254I**
M8a (*β* & *ω* = 1)	*p_0_* = 0.38, *p* = 2.18, *q* = 16.36, *p_1_* = 0.62, *ω_1_* = 1.00	−6190.38	*M8* versus *M8a* 71.20 (**3.23E‐17**)	

^a^ *p* and *q* denote the parameters for beta distribution.

^b^ Amino acid sites estimated to be subject to positive selection are listed.

^c^ The position is referred to human T2R1. Positively selected sites identified in both M2a and M8 were labeled with boldface in the result of M8.

To make clear whether the selective pressure of different regions of *T2R1* were different, we performed the model comparison of M0 (*ω* = 1) and M0 (one *ω*), the result is shown in Table 2.

**TABLE 2:** Selective pressure analyses of different regions in *T2R1* gene

Structure	LnL(*ω* = 1)	LnL(one *ω*)	*ω*	2dl	*p* value (*df* = 1)
ECs	−2253.23	−2240.31	1.97	25.84	**3.71E‐07**
TMs	−2753.21	−2743.60	0.62	19.22	**1.16E‐05**
ICs	−1124.97	−1121.27	0.61	7.40	**6.52E‐03**
TM1237	−1424.60	−1417.76	0.57	13.68	**2.17E‐04**
EL1	−724.92	−718.32	2.35	13.20	**2.80E‐04**
EL2	−937.30	−934.04	1.73	6.52	**0.01**

*p* value bigger than 0.05 is marked in bold.

First of all, as the Table 2 showed, *ω* of the three main regions (ECs, TMs, ICs) were different, and the trend was ECs > TMs>ICs. Secondly, LTR between Model 0 (*ω* = 1) and Model 0 (one *ω*) revealed that, compared to null model Model 0 (*ω* = 1), Model 0 (one *ω*) fitted the data of ECs, ICs, TM1237, EL1, and EL2 significantly better because all the *p* values were smaller than 0.05, indicating that TMs, ICs, and TM1237 were under purifying selection (*ω* < 1) while ECs, EL1, and EL2 were subject to positive selection (*ω* > 1). Finally, the *ω* value of ECs, EL1, and EL2 was 1.97, 2.35, and 1.73, respectively, significantly bigger than 1, suggestive of strong positive selection acting on these regions.

We further tested the positively selected sites in ECs, EL1, and EL2, and the results are shown in Table 3. In this section, M2a and M8 fitted our data better than other models, no matter in ECs, EL1, or EL2, and 18 (in ECs), 4 (in EL1), and 7 (in EL2) positively selected sites identified in M2a were also found in M8, suggestive of strongly positive selection presence in these areas.

**TABLE 3:** Selective pressure analyses of ECs, EL1 and EL2

Model	Parameter estimates^a^	LnL	2 dl (*P* value)	Positively selected sites^b^
ECs				
M0 (*ω* = 1)	*ω* = 1	−2253.23	*M0 (one ω)* versus *M0 (ω* = *1)* 25.84 (**3.71E‐07**)	
M0 (one *ω*)	*ω* = 1.97	−2240.31		None
M1a (nearly neutral)	*p_0_* = 0.13, *ω_0_* = 0.23, *p_1_* = 0.87, *ω_1_* = 1.00	−2248.56	*M2a* versus *M1a* 56.16 (**6.38E‐13**)	
M2a (positive selection)	*p_0_* = 0.02, *ω_0_* = 0.00, *p_1_* = 0.48, *ω_1_* = 1.00, *p_2_* = 0.51, *ω_2_* = 3.37	−2220.48		20 M, 23A, 26A, 28L, 29L, 37A, 38G, 39F, 43Y, 47K, 56T, 57L, 59I, 62F, 70L, 74I, 78I, 82F
M7 (*β*)	*p* = 0.16, *q* = 0.01	−2248.65	*M8* versus *M7* 56.38 (**5.72E‐13**)	Not allowed
M8 (*β* & *ω* > 1)	*p_0_* = 0.48, *p* = 0.17, *q* = 0.01, *p_1_* = 0.52, *ω_1_* = 3.29	−2220.46		8V, **20 M**, **23A**, **26A**, **28L**, **29L**, **37A**, **38G**, **39F**, **43Y**, **47K**, **56T**, **57L**, **59I**, **62F**, 66A, **70L**, **74I**, **78I**, **82F**
M8a (*β* & *ω* = 1)	*p_0_* = 0.13, *p* = 29.75, *q* = 99.00, *p_1_* = 0.87, *ω_1_* = 1.00	−2248.58	*M8* versus *M8a* 56.24 (**6.41E‐14**)	
EL1				
M0 (*ω* = 1)		−724.92	*M0 (one ω)* versus *M0* *(ω* = *1)* 13.20 (**2.80E‐04**)	
M0 (one *ω*)	2.35	−718.32		None
M1a (nearly neutral)	*p_0_* = 1E‐5, *ω_0_* = 0.51, *p_1_* = 1.00, *ω_1_* = 1.00	−724.92	*M2a* versus *M1a* 31.34 (**1.57E‐07**)	
M2a (positive selection)	*p_0_* = 0.45, *ω_0_* = 1.00, *p_1_* = 0.24, *ω_1_* = 1.00, *p_2_* = 0.30, *ω_2_* = 4.89	−709.25		13 M, 16A, 21L, 22L
M7 (*β*)	*p* = 65.53, *q* = 0.005	−724.92	*M8* versus *M7* 31.34 (**1.57E‐07**)	Not allowed
M8 (*β* & *ω* > 1)	*p_0_* = 0.70, *p* = 65.45, *q* = 0.005, *p_1_* = 0.30, *ω_1_* = 4.89	−709.25		**13 M**, **16A**, 19A, **21L**, **22L**
M8a (*β*& *ω* = 1)	*p_0_* = 1E‐5, *p* = 4.99, *q* = 0.55, *p_1_* = 1.00, *ω_1_* = 1.00	−724.92	*M8* versus *M8a* 31.34 (**2.17E‐08**)	
EL2				
M0 (*ω* = 1)	*ω* = 1	−937.30	*M0 (one ω)* versus *M0 (ω* = *1)* 6.52 (**0.01**)	
M0 (one *ω*)	*ω* = 1.73	−934.04		None
M1a (nearly neutral)	*p_0_* = 0.05, *ω_0_* = 0.00, *p_1_* = 0.95, *ω_1_* = 1.00	−934.70	*M2a* versus *M1a* 10.90 (**0.004**)	
M2a (positive selection)	*p_0_* = 0.05, *ω_0_* = 0.00, *p_1_* = 0.33, *ω_1_* = 1.00, *p_2_* = 0.62, *ω_2_* = 2.32	−929.25		4A, 5G, 6F, 10Y, 14K, 23T, 24L
M7 (*β*)	*p* = 0.05, *q* = 0.005	−935.16	*M8* versus *M7* 9.50 (**0.009**)	Not allowed
M8 (*β* & *ω* > 1)	*p_0_* = 0.06, *p* = 0.005, *q* = 3.69, *p_1_* = 0.94, *ω_1_* = 1.88	−930.41		**4A**, **5G**, **6F**, **10Y**, **14K**, **23T**, **24L**, 26I, 29F
M8a (*β* & *ω* = 1)	*p_0_* = 0.05, *p* = 0.005, *q* = 2.50, *p_1_* = 0.95, *ω_1_* = 1.00	−934.70	*M8* versus *M8a* 8.58 (**0.003**)	

^a^ *p* and *q* denote the parameters for beta distribution.

^b^ Amino acid sites estimated to be subject to positive selection are listed.

^c^ The position is referred to human T2R1. Positively selected sites identified in both M2a and M8 were labeled with boldface in the result of M8.

When comparing the positively selected sites between Tables 1 and 3, we found that both tables supported 77 M, 80A, 85L, 86L of EL1, and 150F, 158K, 167T, 168L of EL2 as the positively selected sites, and these sites were marked in Figure 2.

## 4 DISCUSSION

In this study, we explored the evolutionary forces shaping *T2R1* evolution in 35 primates and found that *T2R1* was under neutral evolution (*ω* = 0.94; *p* value = 0.41), conforming to the Fischer et al. (2005) which suggested that the range for *ω* value of bitter receptor genes was from 0.43 to 1.58 and the average value of *ω* was 0.93. The result was different from the *T2R38* which was subject to purifying selection (*ω* = 0.60; *p* = 2.77 × 10^−9^) in a previous study (Wooding, 2011), suggesting the trend of purifying selection is not universal among *T2R*s. Although the *ω* for *T2R1* is not significantly different from 1, it does not mean all the regions of T2R1 are under neutrality, because this result can easily arise when different types of selection act on different parts of the protein. Furthermore, different sites in most (if not all) proteins serve different functions and have different intra and intermolecular interactions, so it is expected that they will be under different types of selection at different times in the protein's history. In our study, three pairs of models (M1a vs. M2a, M7 vs. M8, and M8a vs. M8) were used to detect the positively selected sites, and the results showed that all three pairs of models rejected the null hypothesis and chose the alternative hypothesis. That is, *T2R1* has positively selected sites, and many sites with higher posterior probability (PP) were identified. By contrast, no high PP positively selective sites of *T2R38* were identified in either M2a or M8 (Wooding, 2011). Taken together, *T2R1* was subject to strong positive selection during the evolutionary process of primates, and further, most positively selected sites are distributed in extracellular regions, which are predicted to be able to recognize and bind with ligands (Adler et al., 2000). Selective pressure analyses on different regions of *T2R1* showed that extracellular loops (ECs) had strong signs of adaptive evolution, while both TMs and ICs were subject to purifying selection. Further analysis revealed that both EL1and EL2 were under positive selection, with 30% (*ω* = 4.89) and 62% (*ω* = 2.32) sites under positive selection in EL1 and EL2, respectively, and the *ω* values for ECs, the gene regions which directly involved in ligand recognition, were high, which is probably due to the large amount of ligands that T2R1 responds to and the rapid substitution of ECs is aimed at accommodating to various of bitter tastants. In contrast with T2R38, which is highly sensitive to compounds synthesized by the worldwide distributed cruciferous plants, its EL2 region responds to bitter tastant directly; thus, such region is subject to positive selection (Wooding, 2011). Besides, molecular modeling showed that the ligand binding pocket of T2R1 exists in the extracellular region of the receptor, and the extracellular loops 1 and 2 on the binding pocket form a cap‐like structure (Upadhyaya et al., 2010), which further provided the structure foundation for rapid change of EL1 and EL2. In regard to TMs, which have dual roles—forming a binding pocket for bitter tastants recognition (Upadhyaya et al., 2010) and transmitting binding signal to intracellular regions interacted with G proteins (Okada et al., 2001), the results in our study suggested that TM1237 were constrained, which may be attributed to the need of maintaining basic functionality for binding signal transmission, just as it is suggested in T2R38 (Wooding, 2011).

In summary, our results approximately agreed with the previous research and verified the long‐standing hypothesis that different selective pressures have acted on ECs, TMs, and ICs, with ECs being unstable while TMs and ICs constrained (Strotmann et al., 2011). In *T2R1*, ECs have higher *d*_N_/*d*_S_ than that of TMs and ICs, and it is worthy to note that, positive selection is imposed on extracellular regions especially EL1 and EL2, which may be related to their involvement of forming a cap‐like structure that participating in bitter tastants binding. Furthermore, during the evolution of primates, different functional regions of the *T2R1* gene are under different selective pressures, which may lead to functional divergence and specialization, and ultimately contributes to the adaptation of feeding ecology to the versatile environment during the evolutionary course of primates. The T2Rs evolution has been tested in many aspects and our study explored it from the view of structure.

At last, the existence of some sites that show evidence of positive selection could be due to many other reasons. The positive selection imposed on EC regions could be attributed to their involvement in tastant recognition, while acknowledging that with certain still need further functional assay. Moreover, some problems with inferences of positive selection based on comparing the models implemented in PAML have been identified, and admittedly most of these problems have been found in tests that involve branch‐site models (Potter et al., 2021; Venkat et al., 2018), but it is not clear how they may translate to the site models. Thus, it is suggested that caution should be taken when we draw conclusions from models comparison by using PAML software package.

## CONFLICT OF INTEREST

The authors have no conflicts of interest to declare.

## AUTHOR CONTRIBUTION

**Xiaoyan Dong:** Data curation (supporting); Formal analysis (supporting); Writing‐original draft (supporting). **Qiufang Liang:** Data curation (supporting); Supervision (supporting). **Jiaping Li:** Data curation (supporting); Formal analysis (supporting); Supervision (supporting). **Ping Feng:** Formal analysis (equal); Supervision (equal); Writing‐original draft (lead).

## Supporting information

Supplementary Material

## Data Availability

All relevant data are presented in the paper and the supplementary materials.
