# Supplementary material for: Positive selection drives the evolution of a primate bitter taste receptor gene
Source: Ecol Evol. 2021 Mar 23;11(10):5459–67. doi: 10.1002/ece3.7440 (PMC8131804; doi:10.1002/ece3.7440)
Supplement: Supplementary file 1 — Supplementary Material [file ECE3-11-5459-s001.docx]

Table S1 Information of genomes used in this study.

| Family | Species name | Assembly ID | Contig N50 (kbp) |
| --- | --- | --- | --- |
| Cercopithecidae | *Macaca mulatta* | 2345051 | 46,609 |
|  | *Macaca fascicularis* | 704988 | 86 |
|  | *Macaca nemestrina* | 313501 | 107 |
|  | *Macaca fuscata* | 1696501 | 95 |
|  | *Papio anubis* | 4779671 | 1,465 |
|  | *Theropithecus gelada* | 1767811 | 310 |
|  | *Mandrillus leucophaeus* | 311021 | 31 |
|  | *Cercocebus atys* | 313491 | 113 |
|  | *Chlorocebus sabaeus* | 132581 | 90 |
|  | *Piliocolobus tephrosceles* | 5385541 | 98 |
|  | *Trachypithecus francoisi* | 5396161 | 16,183 |
|  | *Colobus angolensis* | 311101 | 38 |
|  | *Pygathrix nemaeus* | 2210621 | 51 |
|  | *Nasalis larvatus* | 221311 | 13 |
|  | *Rhinopithecus bieti* | 777531 | 36 |
|  | *Rhinopithecus roxellana* | 4120761 | 5,724 |
| Hylobatidae | *Nomascus leucogenys* | 3590591 | 12,783 |
| Hominoidea | *Pongo abelii* | 1529631 | 11,074 |
|  | *Pongo pygmaeus* | 1510921 | 17 |
|  | *Gorilla gorilla* | 4439481 | 9,523 |
|  | *Pan troglodytes* | 1642151 | 12,269 |
|  | *Pan paniscus* | 474211 | 67 |
| Cebidae | *Callithrix jacchus* | 7157801 | 25,227 |
|  | *Aotus nancymaae* | 1130511 | 126 |
|  | *Saimiri boliviensis* | 420378 | 39 |
|  | *Cebus capucinus* | 716501 | 41 |
| Tarsiidae | *Tarsius syrichta* | 64501 | 38 |
| Galagidae | *Otolemur garnettii* | 396188 | 27 |
| Daubentoniidae | *Daubentonia madagascariensis* | 2211621 | 299 |
| Indriidae | *Propithecus coquereli* | 315741 | 28 |
| Cheirogaleidae | *Microcebus murinus* | 998221 | 211 |
| Lemuridae | *Prolemur simus* | 1769311 | 48 |
|  | *Eulemur flavifrons* | 469021 | 27 |
|  | *Eulemur macaco* | 469011 | 20 |

Table S2 *T2R1* gene sequences identified in this study.

>Macaca mulatta

ATGCTAGAGTCACACCTGATTATCTATTTTCTTTTTGCAGTGATACAATTTCTTCTTGGGACTTTCACGAATGGCATCATTGTGGTGGTGAATGGCATTGACTTGATCAAGCACAGAAAAATGGCTCCGCTGGATCTTCTTCTTTCTTGCCTGGCGGTTTCTAGAATTTTTCTGCAGTTGTTCATCTTCTACATTAATGTGGTTGTTATCTTCTTGATAGAATTCATCACGTGTTCTGCGAGTTGTGCATTTATCGTATTTGTAAATGAATTGGAACTTTGGCTTGCCACATGGCTCGGCGTTTTCTACTGTGCCAAGGTTGCCAGCGTCCCTCACCCACTCTTCGTCTGGTTGAAGATGAGGATATCCAAGTTAGTCCCGTGGATGATCCTGGGGTCTCTGCTATATGTATCTGTGATTTGTATTTTCCATAGCAAATATACAGGGTTTATGGTCCCGTACTTCTTAAGGAACCTTTTCTTCCAAAATGCCACAATTCAAATAGAAGTTAAACAGGCTATACAGATTTTCTCTTTTGTTGCTGAGCTCTTAGTGCCATTACTTATCTTCCTTGTTGCTGTTCTGCTCTTGATTTTCTCTCTGGGGAGGCACACCCGGCAAATGAGAAACACAGTGGCTGGCAGCAGGGTTCCTGGCAGGGGTGCCCACATCAGCGCGTTGCTGTCCATCCTGTCCTTCCTGATCCTCTACATCTCCCACTACCTGATAAAAGCTTTTCTCTCTTCTCTAAAGTTTCATGTCAAAAGGTTCGTCTTTCTGTTCTGCATCCTTGTGATTGGTACATACCCTTCTGGACACTCTCTCATCTTAATTTTAGGAAATCCTAAATTGAAACAAAATACAAAAGAGTTCCTCTGCCACAGTAAGTGCTGTCAGTGA

>Macaca fascicularis

ATGCTAGAGTCACACCTGATTATCTATTTTCTTTTTGCAGTGATACAATTTCTTCTTGGGACTTTCACGAATGGCATCATTGTGGTGGTGAATGGCATTGACTTGATCAAGCACAGAAAAATGGCTCCGCTGGATCTTCTTCTTTCTTGCCTGGCGGTTTCTAGAATTTTTCTGCAGTTGTTCATCTTCTACATTAATGTGGTTGTTATCTTCTTGATAGAATTCATCACGTGTTCTGCGAGTTGTGCATTTATCGTATTTGTAAATGAATTGGAACTTTGGCTTGCCACATGGCTCGGCGTTTTCTACTGTGCCAAGGTTGCCAGCGTCCCTCACCCACTCTTCGTCTGGTTGAAGATGAGGATATCCAAGTTAGTCCCGTGGATGATCCTGGGGTCTCTGCTATATGTATCTGTGATTTGTATTTTCCATAGCAAATATACAGGGTTTATGGTCCCGTACTTCTTAAGGAACCTTTTCTTCCAAAATGCCACAATTCAAATAGAAGTTAAACAGGCTATACAGATTTTCTCTTTTGTTGCTGAGCTCTTAGTGCCATTACTTATCTTCCTTGTTGCTGTTCTGCTCTTGATTTTCTCTCTGGGGAGGCACACCCGGCAAATGAGAAACACAGTGGCTGGCAGCAGGGTTCCTGGCAGGGGTGCCCACATCAGCGCGTTGCTGTCCATCCTGTCCTTCCTGATCCTCTACATCTCCCACTACCTGATAAAAGCTTTTCTCTCTTCTCTAAAGTTTCATGTCAAAAGGTTCGTCTTTCTGTTCTGCATCCTTGTGATTGGTACATACCCTTCTGGACACTCTCTCATCTTAATTTTAGGAAATCCTAAATTGAAACAAAATACAAAAGAGTTCCTCTGCCACAGTAAGTGCTGTCAGTGA

>Macaca nemestrina

ATGCTAGAGTCACACCTGATTATCTATTTTCTTTTTGCAGTGATACAATTTCTTCTTGGGACTTTCACGAATGGCATCATTGTGGTGGTGAATGGCATTGACTTGATCAAGCACAGAAAAATGGCTCCGCTGGATCTTCTTCTTTCTTGCCTGGCGGTTTCTAGAATTTTTCTGCAGTTGTTCATCTTCTACATTAATGTGGTTGTTATCTTCTTGATAGAATTCATCACGTGTTCTGCGAGTTGTGCATTTATCGTATTTGTAAATGAATTGGAACTTTGGCTTGCCACATGGCTCGGCGTTTTCTACTGTGCCAAGGTTGCCAGCGTCCCTCACCCATTCTTCGTCTGGTTGAAGATGAGGATATCCAAGTTAGTCCCGTGGATGATCCTGGGGTCTCTGCTATATGTATCTGTGATTTGTATTTTCCATAGCAAATATACAGGGTTTATGGTCCCGTACTTCTTAAGGAACCTTTTCTTCCAAAATGCCACAATTCAAATAGAAGTTAAACAGGCTATACAGATTTTCTCTTTTGTTGCTGAGCTCTTAGTGCCATTACTTATCTTCCTTGTTGCTGTTCTGCTCTTGATTTTCTCTCTGGGGAGGCACACCCGGCAAATGAGAAACACAGTGGCTGGCAGCAGGGTTCCTGGCAGGGGTGCCCACATCAGCGCGTTGCTGTCCATCCTGTCCTTCCTGATCCTCTACATCTCCCACTACCTGATAAAAGCTTTTCTCTCTTCTCTAAAGTTTCATGTCAAAAGGTTCGTCTTTCTGTTCTGCATCCTTGTGATTGGTACATACCCTTCTGGACACTCTCTCATCTTAATTTTAGGAAATCCTAAATTGAAACAAAATACAAAAGAGTTCCTCTGCCACAGTAAGTGCTGTCAGTGA

>Chlorocebus sabaeus

ATGCTAGAGTCACACCTGATTATCTATTTTCTTCTTGCAGTGATACAATTTCTTCTTGGGACTTTCACAAATGGCATCATTGTGGTGGTGAATGGCATTGACTTGATCAAGCACAGAAAAATGGCTCCGCTGGATCTTCTTCTTTCTTGCCTGGCGGTTTCTAGAATTTTTCTGCAGTTGTTCATCTTCTACATTAATGTGGTTGTTATCTTCTTGATAGAATTCATCACGTGTTCTGCGAGTTGTGCATTTCTCGTATTTGTAAATGAATTGGAACTTTGGCTTGCCACATGGCTCGGCGTTTTCTACTGTGCCAAGGTTGCCAGCGTCCCTCACCCACTCTTCATCTGGTTGAAGATGAGGATATCCAAGTCAGTCCCGTGGATGATCCTGGGATCTCTGCTATATGTATCTATGATTTGTATTTTCCATATCAAATATACAGGGTTTATGGTCCCGTACTTCTTAAGGAACCTTTTCTTCCAAAATGCCACAATTCAAACAGAAGTTAAACAGGCTATACAGATTTTCTCTTTTGTTGCTGAGCTCTTAGTGCCATTACTTATCTTCCTTGTTGCTGTTCTGCTCTTGATTTTCTCTCTGGGGAGGCACACCCGGCAAATGAGAAACACAGTGGCTGGCAGCAGGGTTCCTGGCAGGGGTGCCCACATCAGCGCGTTGCTGTCCATCCTGTCCTTCCTGATCCTCTACATCTCCCACTACTTGATAAAAACTTTTCTCTCTTCTCTAAAGTTTCATGTCAAAAGGTTCGTCTTTCTGTTCTGCATCCTTGTGATTGGTACATACCCTTCTGGACACTCTCTCATCTTAATTTTAGGAAATCCTAAATTGAAACAAAATACAAAAAAGTTCCTCTGCCACAGTAAGTGCTGTCAGTGA

>Cercocebus atys

ATGCTAGAGTCACACCTGATTATCTCTTTTCTTTTTGCAGTGATACAATTTCTTCTTGGGACTTTCACGAATGGCATCATTGTGGTGGTGAATGGCATTGACTTGATCAAGCACAGAAAAATGGCTCCACTGGATCTTCTTCTTTCTTGCCTGGCGGTTTCTAGATTTTTTCTGCAGTTGTTCATCTTCTACATTAATGTGGTTGTTATCTTCTTGATAGAATTCATCACGTGTTCTGCGAGTTGTGCATTTCTCGTATTTGTAAATGAATTGGAACTTTGGCTTGCCACATGGCTCGGCATTTTCTACTGTGCCAAGGTTGCCAGCGTCCCTCACCCACTCTTCATCTGGTTGAAGATGAGGATATCCAAGTTAGTCCCGTGGATGATCCTGGGGTCTCTGCTATATGTATCTATGATTTGTATTTTCCATAGCAAATATACAGGGTTTATGGTCCCGTACTTCTTAAGGAACCTTTTCTTCCAAAATGCCACAATTCAAACAGAAGTTAAACAGGCTATACAGATTTTCTCTTTTGTTGCTGAGCTCTTAGTGCCATTACTTATCTTCCTTGTTGCTGTTCTGCTCTTGATTTTCTCTCTGGGGAGGCATACCCGGCAAATGAGAAACACAGTGGCTGGCAGCAGGGTTCCTGGCAGGGGTGCCCACATCAGCGCGTTGCTGTCTATCCTGTCCTTCCTGATCCTCTACATCTCCCACTACTTGATAAAAGCTTTTCTCTTTTCTCTAAAGTTTCATGTCAAAAGGTTCGTCTTTCTGTTCTGCATCCTTGTGATTGGTACATACCCTTCTGGACACTCTCTCATCTTAATTTTAGGAAATCCTAAATTGAAACAAAATACAAAAAAGTTCCTCTGCCACAGAAAGTGCTGTCAGTGA

>Mandrillus leucophaeus

ATGCTAGAGTCACACCTGATTATCTCTTTTCTTTTTGCAGTGATACAATTTCTTCTTGGGACTTTCACGAATGGCATCATTGTGGTGGTGAATGGCATTGACTTGATCAAGCACAGAAAAATGGCTCCACTGGATCTTCTTCTTTCTTGCCTGGCGGTTTCTAGATTTTTTCTGCAGTTGTTCATCTTCTACATTAATGTGGTTGTTATCTTCCTGATAGAATTCATCACGTGTTCTGCGAGTTGTGCATTTCTCGTATTTGTAAATGAATTGGAACTTTGGCTTGCCACATGGCTCGGCATTTTCTACTGTGCCAAGGTTGCCAGCGTCCCTCACCCACTCTTCATCTGGTTGAAGATGAGGATATCCAAGTTAGTCCCGTGGATGATCCTGGGGTCTCTGCTATATGTATCTGTGATTTGTATTTTCCATAGCAAATATACAGGGTTTATGGTCCCGTACTTCTTAAGGAACCTTTTCTTCCAAAATGCCACAATTCAAACAGAAGTTAAACAGGCTATACAGATTTTCTCTTTTGTTGCTGAGCTCTTAGTGCCATTACTTATCTTCCTTGTTGCTGTTCTGCTCTTGATTTTCTCTCTGGGGAGGCATACCCGGCAAATGAGAAACATGGTGGCTGGCAGCAGGGTTCCTGGCAGGGGTGCCCACATCAGCGCGTTGCTGTCCATCCTGTCCTTCCTGATCCTCTACATCTCCCACTACTTGATAAAAGCTTTTCTCTTTTCTCTAAAGTTTCATGTCAAAAGGTTCATCTTTCTGTTCTGCATCCTTGTGATTGGTACATACCCTTCTGGACACTCTCTCATCTTAATTTTAGGAAATCCTAAATTGAAACAAAATACAAAAAAGTTCCTCTGCCACAGTAAGTGCTGTCAGTGA

>Papio anubis

ATGCTAGAGTCACACCTGATTATCTATTTTCTTTTTGCAGTGATACAATTTCTTCTTGGGACTTTCACGAATGGCATCATTGTGGTGGTGAATGGCATTGACTTGATCAAGCACAGAAAAATGGCTCCGCTGGATCTTCTTCTTTCTTGCCTGGCGGTTTCTAGAATTTTTCTGCAGTTGTTCATCTTCTACATTAATGTGGTTGTTATCTTCTTGATAGAATTCATCACGTGTTCTGCGAGTTGTGCATTTCTCGTATTTGTAAATGAATTGGAACTTTGGCTTGCCACATGGCTCGGCATTTTCTACTGTGCCAAGGTTGCCAGCGTCCCTCACCCACTCTTCATCTGGTTGAAGATGAGGATATCCAAGTTAGTCCCGTGGATGATCCTGGGGTCTCTGCTATATGTATCTATGATTTGTATTTTCCATAGCAAATATACAGGGTTTATGGTCCCGTACTTCTTAAGGAACCTTTTCTTCCAAAATGCCACAATTCAAACAGAAGTTAAACAGGCTATACAGATTTTCTCTTTTGTTGCTGAGCTCTTAGTGCCATTACTTATCTTCCTTGTTGCTGTTCTGCTCTTGATTTTCTCTCTGGGGAGGCATACCCGGCAAATGAGAAACACAGTGGCTGGCAGCAGGGTTCCTGGCAGGGGTGCCCACATCAGCGCATTGCTGTCCATCCTGTCCTTCCTGATCCTCTACATCTCCCACTACTTGATAAAAGCTTTTCTCTTTTCTCTAAAGTTTCATGTCAAAAGGTTCATCTTTCTGTTCTGCATCCTTGTGATTGGTACATACCCTTCTGGACACTCTCTCATCTTAATTTTAGGAAATCCTAAATTGAAACAAAATACAAAAAAGTTCCTCTGCCACAGAAAGTGCTGTCAGTGA

>Theropithecus gelada

ATGCTAGAGTCACACCTGATTATCTATTTTCTTTTTGCAGTGATACAATTTCTTCTTGGGACTTTCACGAATGGCATCATTGTGGTGGTGAATGGCATTGACTTGATCAAGCACAGAAAAATGGCTCCGCTGGATCTTCTTCTTTCTTGCCTGGCGGTTTGTAGAATTTTTCTGCAGTTGTTCATCTTCTACATTAATGTGGTTGTTATCTTCTTGATAGAATTCATCACGTGTTCTGCGAGTTGTGCATTTCTCATATTTGTAAATGAATTGGAACTTTGGCTTGCCACATGGCTCGGCATTTTCTACTGTGCCAAGGTTGCCAGCGTCCCTCACCCACTCTTCATCTGGTTGAAGATGAGGATATCCAAGTTAGTCCCGTGGATGATCCTGGGGTCTCTGCTATATGTATCTATGATTTGTATTTTCCATAGCAAATATACAGGGTTTATGGTCCCGTACTTCTTAAGGAACCTTTTCTTCCAAAATGCCACAATTCAAACAGAAGTTAAACAGGCTACACAGATTTTCTCTTTTGTTGCTGAGCTCTTAGTGCCATTACTTATCTTCCTTGTTGCTGTTCTGCTCTTGATTTTCTCTCTGGGGAGGCATACCCGGCAAATGAGAAACACAGTGGCTGGCAGCAGGGTTCCTGGCAGGGGTGCCCACATCAGCGCGTTGCTGTCCATCCTGTCCTTCCTGATTCTCTACATCTCCCACTACTTGATAAAAGCTTTTCTCTTTTCTCTAAAGTTTCATGTCAAAAGGTTCATCTTTCTGTTCTGCATCCTTGTGATTGGTACATACCCTTCTGGACACTCTCTCATCTTAATTTTAGGAAATCCTAAATTGAAACAAAATACAAAAAAGTTCCTCTGCCACAGAAAGTGCTGTCAGTGA

>Piliocolobus tephrosceles

ATGCTAGAGTCACACCTGATTATCTATTTTCTTCTTGCAGTGATACAATTTCTTTTTGGGACTTTAACAAATGGCATCATTGTGGTGGTGAATGGCATTGACTTGATCAAGCACAGAAAAATGGCTCCGCTGGATCTTCTTCTTTCTTGCCTGGCGGTTTCTAGAATTTTTCTGCAGTTGTTCATCTTCTACATTAATGTGGTTGTTATCTTCTTGATGGAAATCACCACGTGTTCTGCGAATTGTGTATTTCTCATATTTGTAAATGAATTGGAACTTTGGCTTGCCACATGGCTCGGCGTTTTCTACTGTGCCAAGGTTGCCAGCGTCCCTCACCCACTCTTCATCTGGTTGAAGATGAGGATATCCAAGTTAGTCCCGTGGATGATCCTGGGGTCTCTGGTATATGTATCTATGATTTGTATTTTCCATAGCAAATATACAGGGTTTATGGTCCCATATTTCTTAAGAAACTTTTTCTTCCAAAATGTCACAATTCAAACAGAAGCTAAACCGGGTATACAGATTTTCTCTTTTGTTGCTGAGCTCTTAGTGCCATTACTTATCTTCCTTGTTGCTGTTCTGCTCTTGATTTTCTCTCTGGGGAGGCACTCCCGGCAAATGAGAAACACAGTGGCTGGCAGCAGGGTTCCTGGCAGGGGTGCCTACATCAGCGCGTTGCTGTCCATCCTGTCCTTTCTGATCCTCTACATCTCCCACTACTTGATAAAAACTTTTCTCTCTTCTATAAAGTTTCATGTCAAAAGGTCCATCTTTCTGTTCTGCATCCTTGTGATTGGTACGTACCCTTCTGGACACTCTCTCATCTTAATTTTAGGAAATCCTAAATTGAAACAAAATACAAAAAAGTTCCTCTGCCACAGTAAGTGCTGTCAGTGA

>Nasalis larvatus

ATGCTAGAGTCACACCTGATTATCTATTTTCTTCTTGCAGTGATACAATTTCTTTTTGGGACTTTCACAAATGGCATCATTGTGGTGGTGAATGGCATTGACTTGATCAAGCACAGAAAAATGGCTCCGCTGGATCTTCTTCTTTCTTGCCTGGCGGTTTCTAGAATTTTTCTGCAGTTGTTCATCTTCTACATTGATGTGGTTGTTATCTTCTTGATGGAATTCATCATGTGTTCTGCGAATTGTGTATTTATCATATTTGTAAATGAATTGGAACTTTGGTTTGCCACATGGCTCGGCGTTTTCTACTGTGCCAAGGTTGCCAGCGTCCCTCACCCACTCTTCATCTGGTTGAAGATGAGGATATCCAAGTTAGTCCCGTGGATGATCCTGGGGTCTCTGCTATATGTATCTATGATTTGTATTTTCCGTAGCAAATATACAGGGTTTATGGTCCCATACTTCTTAAGAAACTTTTTCTTCCAAAATGCCACAATTCAAACAGAAGTTAAACGGGGTATACAGATTTTCTCTTTTGTTGCTGAGCTCTTAGTGCCATTACTTATCTTCCTTGTTGCTGTTCTGCTCTTGATTTTCTCTCTGGGGAGGCACTCCCAGCAAATGAGAAACACAGTGGCTGGCAGCAGGGTTCCTGGCAAGGGTGCCCACATCAGCGCGTTGCTGTCCATCCTGTCCTTCCTGATCCTCTACATCTCCCACTACTTGATAAAAGCTTTTCTCTCTTCTCTAAAGTTTCATGTCAAAAGGTCCATCTTTCTATTCTGCATGCTTGTGATTGGTACATACCCTTCTGGACACTCTCTCATCTTAATTTTAGGAAATCCTAAATTGAAACAAAATACAAAAAAGTTCCTCTGCCACAGTAAGTGCTGTCAGTGA

>Trachypithecus francoisi

ATGCTAGAGTCACACCTGATTATCTACTTTCTTCTTGCAGTGATACAATTTCTTTTTGGGACTTTCACAAATGGCATCATTGTGGTGGTGAATGGCATTGACTTGATCAAGCACAGAAAAATGGCTCCGCTGGATCTTCTTCTTTCTTGCCTGGCGGTTTCTAGAATTTTTCTGCAGTTGTTCATCTTCTACATTAATGTGGTTGTTATCTTCTTGATGGAATTCATCACATGTTCTGCGAATTGTGTATTTCTCATATTTGTAAATGAATTGGAACTTTGGCTTGCCACATGGCTCGGCGTTTTCTACTGTGCCAAGGTTGCCAGCGTCCCTCACCCACTCTTCATCTGGTTGAAGATGAGGATATCCAAGTTAGTCCCATGGATGATCCTGGGGTCTCTGCTATATGTATCTATGATTTGTATTTTCCATAGCAAATATACAGGGCTTATGGTCCCATACTTCTTAAGAAACTTTTTCTTCCAAAATGCCACAATTCAAACAGAAGTTAAACAGGGTATACAGGTTTTCTCTTTTGTTGCTGAGCTCTTAGTGCCATTACTTATCTTCCTTGTTGCTGTTCTGCTCTTGATTTTCTCTCTGGGCAGGCACTCCCGGCAAATGAGAAACACAGTGGCTGGCAGCAGGGTTCCTGGCAGGGGTGCCCACATCAGCGCGTTGCTGTCCATCCTGTCCTTCCTGATCCTCTACATCTCCCACTACTTGATAAAAACTTTTCTCTCTTCCCTAAAGTTTCATGTCAAAAGGTCCATCTTTCTGTTCTGCATCCTTGTGATTGGTACATACCCTTCTGGACACTCTCTCATCTTAATTTTAGGAAATCCTAAATTGAAACAAAATACAAAAAAGTTCCTCTGCCACAGTAAGTGCTGTCAGTGA

>Pygathrix nemaeus

ATGCTAGAGTCACACCTGATTATCTATTTTCTTCTTGCAGTGATACAATTTCTTTTTGGGACTTTCACAAATGGCATCATTGTGGTGGTGAATGGCATTGACTTGATCAAGCACAGAAAAATGGCTCCGCTGGATCTTCTTCTTTCTTGCCTGGCGGTTTCTAGAATTTTTCTGCAGTTGTTCATCTTCTACATTAATGTGGTTGTTATCTTCTTGATGGAATTCATCACGTGTTCTGCGAATTGTGTATTTCTCATATTTGTAAATGAATTGGAACTTTGGCTTGCCACATGGCTCGGCGTTTTCTACTGTGCCAAGGTTGCCAGCGTCCCTCACCCACTCTTCATCTGGTTGAAGATGAGGATATCCAAGTTAGTCCCGTGGATGATCCTGGGGTCTCTGCTATATGTATCTATGATTTGTATTTTCCATAGCAAATATACAGGGTTTATGGTCCCATACTTCTTAAGAAACTTTTTCTTCCAAAATGCCACAATTCAAACAGAAGTTAAACAGGGTATACAGGTTTTCTCTTTTGTTGCTGAGCTCTTAGTGCCATTACTTATCTTCCTTGTTGCTGTTCTGCTCTTGATTTTCTCTCTGGGGAAGCACTCCCGGCAAATGAGAAACACAGTGGCTGGCAGCAGGGTTCCTGGCAGGGGTGCCCACATCAGCGCGTTGCTGTCCATCCTTTCCTTCCTGATCCTCTACATCTCCCACTACTTGATAAAAGCTTTTCTCTCTTCTCTAAAGTTTCATGTCAAAAGGTCCATCTTTCTGTTCTGCATCCTTGTGATTGGTACATACCCTTCTGGACACTCTCTCATCTTAATTTTAGGAAATCCTAAATTGAAACAAAATACAAAAAAGTTCCTCTGCCACAGTAAGTGCTGTCAGTGA

>Rhinopithecus bieti

ATGCTAGAGTCACACCTGATTATCTATTTTCTTCTTGCAGTGATACAATTTCTTTTTGGGACTTTCACAAATGGCATCATTGTGGTGGTGAATGGCATTGACTTGATCAAGCACAGAAAAATGGCTCCGCTGGATCTTCTTCTTTCTTGCCTGGTGGTTTCTAGAATTTTTCTGCAGTTGTTCATCTTCTACATTAATGTGGTTGTTATCTTCTTGATGGAATTTATCACGTGTTCTGCGAATTGTGTATTTCTCATATTTGTAAATGAATTGGAACTTTGGCTTGCCACATGGCTCGGCGTTTTCTACTGTGCCAAGGTTGCCAGCGTCCCTCACCCACTCTTCATCTGGTTGAAGATGAGGATATCCAAGTTAGTCCCGTGGATGATCCTGGGGTCTCTGCTATATGTATCTATGATTTGTATTTTCCATAGCAAATATACAGGGTTTATGGACCCATACTTCTTAAGAAACTTTTTCTTCCAAAATGCCACAATTCAAACAGAAGTTAAACAGGGTATACAGGTTTTCTCTTTTGTTGCTGAGCTCTTAGTGCCATTACTTATCTTCCTTGTTGCTGTTCTGCTCTTGATTTTCTCTCTGGGGAGGCACTCCCGGCAAATGAGAAACACAGTGGCTGGCAGCAGTGTTCCTGGCAGGGGTGCCCACATCAGCGCGTTGCTGTCCATCCTGTCCTTCCTGATCCTCTACATCTCCCACTGCTTGATAAAAGCTTTTCTCTCTTCTCTAAAGTTTCATGTCAAAAGGTCCATCTTTCTGTTCTGCATCCTTGTGATTGGTACATACCCTTCCGGACACTCTCTCATCTTAATTTTAGGAAATCCTAAATTGAAACAAAATACAAAAAAGTTCCTCTGCCACAGTAAGTGCTGTCAGTGA

>Rhinopithecus roxellana

ATGCTAGAGTCACACCTGATTATCTATTTTCTTCTTGCAGTGATACAATTTCTTTTTGGGACTTTCACAAATGGCATAATTGTGGTGGTGAATGGCATTGACTTGATCAAGCACAGAAAAATGGCTCCGCTGGATCTTCTTCTTTCTTGCCTGGTGGTTTCTAGAATTTTTCTGCAGTTGTTCATCTTCTACATTAATGTGGTTGTTATCTTCTTGATGGAATTCATCACGTGTTCTGCGAATTGTGTATTTCTCATATTTGTAAATGAATTGGAACTTTGGCTTGCCACATGGCTCGGCGTTTTCTACTGTGCCAAGGTTGCCAGCGTCCCTCACCCACTCTTCATCTGGTTGAAGATGAGGATATCCAAGTTAGTCCCGTGGATGATCCTGGGGTCTCTGCTATATGTATCTATGATTTGTATTTTCCATAGCAAATATACAGGGTTTATGGACCCATACTTCTTAAGAAACTTTTTCTTCCAAAATGCCACAATTCAAACAGAAGTTAAACAGCGTATACAGGTTTTCTCTTTTGTTGCTGAGCTCTTAGTGCCATTACTTATCTTCCTTGTTGCTGTTCTGCTCTTGATTTTCTCTCTGGGGAGGCACTCCCGGCAAATGAGAAACACAGTGGCTGGCAGCAGTGTTCCTGGCAGGAGTGCCCACATCAGCGCGTTGCTGTCCATCCTGTCCTTCCTGATCCTCTACATCTCCCACTGCTTGATAAAAGCTTTTCTCTCTTCTCTAAAGTTTCATGTCAAAAGGTCCATCTTTCTGTTCTTCATCCTTGTGATTGGTACATACCCTTCCGGACACTCTCTCATCTTAATTTTAGGAAATCCTAAATTGAAACAAAATACAAAAAAGTTCCTCTGCCACAGTAAGTGCTGTCAGTGA

>Nomascus leucogenys

ATGCTAGAGTCTCACCTCGTTATCTATTTTCTTCTTGCAGTGATACAATTTCTTCTTGGGACTTTCACAAATGGCATCATTGTGGTGGTGAACGGCATTGACTTGATCAAGCACAGAAAAATGGCTCCACTGGATCTCCTTCTTTCTTGCCTGGCGGTTTCTAGAATTTTTCTGCAGTTTTTCATCTTCTACGTTAATGTGATTGTTATCTTCTTGATAGAATTCATCAAGTGTTCTACAAATTGTGCAATTCTCTTATTTATAAATGAATTGGAACTTTGGCTTGCCACATGGCTCGGCGTTTTCTACTGTGCCAAGGTTGCCAGCGTCCCTCACCCACTCTTCATCTGGTTGAAGATGAGGATATCCAAGTTGGTCCCATGGATGATCCTGGGGTCTCTGCTATATGTATCTATAATTTGTGTTTTCCATAGCAAATATGCAGGGCTTACGGTCCCATACTTCTTAAGGAACTTTTTATCCCAAAATGCCACGATTCAAAAAGAAGATACACCGGCTATACAGGTTTTCTCTTATGTTGCTGAGTTCTTAGTGCCATTACTTATCTTCCTTGTTGCTGTTCTGCTTTTGATTTTCTCTCTGGGGAGGCACACCCAGCAAATGAGAAACACAGTGGCCGGCAGCAGGGTTCCTGGCAGGGATGCCCCCAGCAGCGCGTTGCTGTCTGTCCTGTCCTTCCTGATCCTCTGCTTCTCCCACTGCATGATAAAAGTTTTTCTCTCTTCTCTAAAGTTTCATGTCAGAAGGTTCATCTTTCTGTTCTTCATCCTTGTGATTGGTATATACCCTTCTGGACACTCTCTCATCTTAATTTTAGGAAATCCTAAATTGAAACAAAATGCAAAAAAGTTCCTCCTCCACAGTAAGTGCTGTCAGTGA

>Gorilla gorilla

ATGCTAGAGTCTCACCTCATTATCTATTTTCTTCTTGCAGTGATACAATTTCTTCTTGGGATTTTCACAAATGGCATCATTGTGGTGGTGAATGGCATTGACTTGATCAAGCACAGAAAAATGGCTCCGCTGGATCTCCTTCTTTCTTGTCTGGCAGTTTCTAGAATTTTTCTGCAGTTGTTCATCTTCTACGTTAATGTGATTGTTATCTTCTTCATAGAATTCATCATGTGTTCTGCGAATTGTGCAATTCTCTTATTTATAAATGAATTGGAACTTTGGCTTGCCACATGGCTCGGCGTTTTCTATTGTGCCAAGGTTGCCAGCGTCCGTCACCCACTCTTCATCTGGTTGAAGATGAGGATATCCAAGCTGGTCCCTTGGATGATCCTGGGGTCTTTGCTATATGTATCTATGATTTGTGTTTTCCATAGCAAATATGCAGGGTTTATGGTCCCATACTTCTTAAGGAACTTTTTCTCCCAAAATACCACAATTCAAAAAGAAGATACACTGGCTATACAGATTTTCTCTTTTGTTGCTGAGTTCTCAGTGCCATTGCTTATCTTCCTTGTTGCTGTTTTGCTCTTGATTTTCTCTCTGGGGAGGCACACCCGGCAAATGAGAAACACAGTGGCCGGCAGCAGGGTTCCTGGCAGAGGTGCACCCATCAGCGCGTTGCTGTCTATCCTGTCCTTCCTGATCCTCTACTTCTCCCACTGCATGATAAAAGTTTTTCTCTCTTCTCTAAAGTTTCACATCAGAAGGTTCATCTTTCTGTTCTTCATCCTTGTGATTGGTATATACCCTTCTGGACACTCTCTCATCTTAATTTTAGGAAATCCTAAATTGAAACAAAATGCAAAAAAGTTCCTCCGCCACAGTAAGTGCTGTCAGTGA

>Pongo abelii

ATGCTAGAGTCTCACCTCATTATCCATTTTCTTCTTGCAGTGATACAATTTCTTCTTGGGACTTTCACAAATGGCATCATTGTGGTGGTGAATGGCATTGACTTGATCAAGCACAGAAAAATGGCTCCGCTGGATCTCCTTCTTTCTTGCCTGGCAGTTTCTAGAATTTTTCTGCAGTTGTTCATCTTCTACGTTAATGTGATTGTTATCTTCTTCATAGAATTCATCATGTGTTCTGAGAATTGTGCAATTCTCTTATTTATAAATGAATTGGAACTTTGGCTTGCCACATGGCTCGGCGTTTTCTACTGTGCCAAGGTTGCCAGCGTGCCTCACCCACTCTTCATCTGGTTGAAGATGAAGATATCCAAGTTGGTCCCATGGATGATCCTGGGATCTCTGCTATATGTATCTATGACTTGTGTTTTCCATAGCAAATATGCAGGGTTTATGGTCCCATACTTCTTAAGGAACTTTTTCTCCCAAAATGCTACAATTCAAAAAGAAGATACACCGGCTATACAGATTTTCTCTTTTGTTGCTGAGTTCTTAGTGCCATTGCTTATCTTCCTTGTTGCTGTTCTGCTCTTGATTTTCTCTCTGGGGAGGCACACCCGGCAAATGAGAAACACAGTGGCCGGCAGCAGGGTTCCTGGCAGGGGTGCCCCCATCAGTGCGTTGCTGTCTATCCTGTCCTTCGTGATCCTCTACTTCTCCCACTGCATGATAAAAGTTTTTCTCTCTTCTCTAAAGTTTCATGTCAGAAGTTTCATCCTTCCATTCTTCATCCTTGTGATTGGTATATACCCTTCTGGACACTCTCTCATCTTAATTTTAGGAAATCCTAAATTGAAACAAAATGCAAAAAAGTTCCTCCTCCACAGTAAGTGCTGTCAGTGA

>Pan troglodytes

ATGCTAGAGTCTCACCTCATTATCTATTTTCTTCTTGCAGTGATACAATTTCTTCTTGGGATTTTCACAAATGGCATCATTGTGGTGGTGAATGGCATTGACTTGATCAAGCACAGAAAAATGGCTCCGCTGGATCTCCTTCTTTCTTGTCTGGCAGTTTCTAGAATTTTTCTGCAGTTGTTCATCTTCTACGTTAATGTGATTGTTATCTTCTTCATAGAATTCATCATGTGTTCTGCGAATTGTGCAATTCTCTTATTTGTAAATGAATTGGAACTTTGGCTTGCCACATGGCTCGGCGTTTTCTATTGTGCCAAGGTTGCCAGCGTCCGTCACCCACTCTTCATCTGGTTGAAGATGAGGATATCCAAGCTGGTCCCATGGATGATCCTGGGGTCTCTGCTATATGTATCTATGATTTGTGTTTTCCATAGCAAATATGCAGGGTTTATGGTCCCACACTTCTTAAGGAACTTTTTCTCCCAAAATGCCACAATTCAAAAAGAAGATACACTGGCTATACAGATTTTCTCTTTTGTTGCTGAGTTCTCAGTGCCATTGCTTATCTTCCTTGTTGCTGTTTTGCTCTTGATTTTCTCTCTGGGGAGGCACACCCGGCAAATGAGAAACACAGTGGCGGGCAGCAGGGTTCCTGGCAGGGGTGCACCCATCAGCGCGTTGCTGTCTATCCTGTCCTTCCTGATCCTCTACTTCTCCCACTGCATGATAAAAATTTTTCTCTCTTCTCTAAAGTTTCACGTCAGAAGGTTCATCTTTCTGTTCTTCATCCTTGTGATTGGTATGTACCCTTCTGGACACTCTCTCATCTTAATTTTAGGAAATCCTAAATTGAAACAAAATGCAAAAAAGTTCCTCCTCCACAGTAAGTGCTGTCAGTGA

>Pan paniscus

ATGCTAGAGTCTCACCTCATTATCTATTTTCTTCTTGCAGTGATACAATTTCTTCTTGGGATTTTCACAAATGGCATCATTGTGGTGGTGAATGGCATTGACTTGATCAAGCACAGAAAAATGGCTCCGCTGGATCTCCTTCTTTCTTGTCTGGCAGTTTCTAGAATTTTTCTGCAGTTGTTCATCTTCTACGTTAATGTGATTGTTATCTTCTTCATAGAATTCATCATGTGTTCTGCGAATTGTGCAATTCTCTTATTTGTAAATGAATTGGAACTTTGGCTTGCCACATGGCTCGGCGTTTTCTATTGTGCCAAGGTTGCCAGCGTCCGTCACCCACTCTTCATCTGGTTGAAGATGAGGATATCCAAGCTGGTCCCATGGATGATCCTGGGGTCTCTGCTATATGTATCTATGATTTGTGTTTTCCATAGCAAATATGCAGGGTTTATGGTCCCACACTTCTTAAGGAACTTTTTCTCCCAAAATGCCACAATTCAAAAAGAAGATACACTGGCTATACAGATTTTCTCTTTTGTTGCTGAGTTCTCAGTGCCATTGCTTATCTTCCTTGTTGCTGTTTTGCTCTTGATTTTCTCTCTGGGGAGGCACACCCGGCAAATGAGAAACACAGTGGCGGGCAGCAGGGTTCCTGGCAGGGGTGCACCCATCAGCGCGTTGCTGTCTATCCTGTCCTTCCTGATCCTCTACTTCTCCCACTGCATGATAAAAGTTTTTCTCTCTTCTCTAAAGTTTCACGTCAGAAGGTTCATCTTTCTGTTCTTCATCCTTGTGATTGGTATATACCCTTCTGGACACTCTCTCATCTTAATTTTAGGAAATCCTAAATTGAAACAAAATGCAAAAAAGTTCCTCCTCCACAGTAAGTGCTGTCAGTGA

>Saimiri boliviensis

ATGCTGGAGTCTGGCCTCATTACCTGCTTTCTTCTTGCAGTGATACAATTTCTTCTTGGGATTTTCATGAATGGCATCATTGTGGTGGTGAACGGTATTGACTTGATCAAGCACAGAAAAATGGCTCCAGTGGATCTCCTTCTTTCCTGCCTGGCACTTTCTAGAACTTTTCTGCAGTTGTTCATCTTCTACATTAATCTGGTTGTTCTCTTCTTGATGAAATTAATCGCGTATCCTGAGAGTTATATAATTATCCTGTTTATAAACGAATTGGAAATTTGGCTTGCCACATGGCTTGGTGTTTTCTACTGCACCAAGGTTTCCAGCGTCCCTCACCCACTCTTCATCTGGTTGAAGATGAAGATATCCAAGTTGGTCCCATGGATGATCCTGGGGTCTCTGCTGTATGTATCTATCATTTGTGTTTTCCGTAGCAGATATTCAGAGTTTTTTGTTCCACACAACCTCATGAACTTTTTCTCTCAAAATGACACAATTCAAATAGAAGATATACCTGCTACACAGATTACCTTTTTTGCTGCTGAGCTCTTAGTGCCACTGCTTATTTTCCTTGCTGCTGTTCTGCTCTTGATTTTTTCTTTGGGGAGGCACACCTGGCAAATGAGAAAGACAGTAGCCCGCAGCAGCGTCCCTGGCAGGAGTGGCCACATGAGCGCCTGGCTGTCCATCCTGTCCTTCCTGATCCTTTATGTTTCTCACTATATGATGAAAGCTTTTCTCTGTTCTATAAGGTTTCACGTCAGAAGGTTCATCTTTATGCTCTGCATCCTTGTGATTGGCACATACCCTTCTGGACACTCTCTCATCTTAATTTTAGGAAATCCTAAATTGAAACAAAATGCAAAAAAGTTCCTCCTCCACAGTAAGTGCTGTCAGTGA

>Cebus capucinus

ATGCTGGAGTCTGACCTCATTATCTGCTTTCTTCTTGCAGTGATACAATTTCTTCTTGGGATTTTCATGAATGGCATCATTGTGGTGGTGAATGGCATTGACTTGATCAAGCACAGAAAAATGGCTCCGGTGGATCTCCTTCTTTCCTGCCTGGCACTTTCTAGAACTTTTCTGCAGTTTTTCATCTTCTACATTAATCTGGTTGCTCTCTTCTTGATGAAATTAATCATGTATCCTGAGAGTTGTGTAATTGTCATGTTTATAAACGAATTGGAACTTTGGCTTGCCACATGGCTTGGCGTGTTCTACTGCGCCAAGGTTTCCAGCGTCCCTCACCCACTCTTCATCTGGTTGAAGATGAAGATATCCAAGTTGGTCCCGTGGATGATCCTGGGGTCTCTGCTATATGTATCTATCATTTGTGTTTTCCATAGCAAATATTCAGTGTGTTTTGTTCCACACAGCCTCATGAACTTTTTCTCCCAAAATGCCACAATTCAAATAGAAGATATACCTGCTATACAGATTACCTCTGTTGCTGCTGAGCTCTTAGTGCCACTGCTTATTTTCCTTGCTGCTGTTCTGCTCTTGATTTTTTCTTTGGGGAGACACGCCTGGCAGATGAGAAACACAGTAGCCAGTAGCAGGGTTCCTGGCAGGAGTGGCCACATCAGCGCCTGGCTGTCCATCCTGTCCTTCCTAATCCTCTATGTTTCCCACTACATGATGAAAGTTTTTCTCTATTCTATAAGGTTTCACATCAGAAGGTTCATCTTTATGCTCTGCATCCTTGTGATTGGCACATACCCTTCTGGACACTCTCTCATCTTAATTTTAGGAAATCCTAAATTGAAACAAAATGCAAAAAAGTTCCTCCTCCACAGTAAGGGCTGTCAGTGA

>Callithrix jacchus

ATGCTGGAGTCTGACCTCATTCTCTGCTTTCTTCTTGCAGTGATGCAGTTTCTTCTTGGAATTTCCATGAATGGCATTATTGTGGTGGTGAATGGCATTGACTTGATCAAGCACAGAAAAATGGCTCCAGTGGATATCCTTCTTTCCTGCCTGGCACTTTCTAGAACTTTCCTGCAGTTGTTTATCTTCTACGTTAATCTGGTTGTTCTCTTCTTGATGAAATTAATCATGTATCCTGAGAGTTTTATAATTATCATGTTTATAAACGAATTGGAACTTTGGTTTGCCACATGGCTTGGCGTTTTCTACTGCACCAAGGTTTCCAGCGTCCCTCACCCACTCTTCATCTGGCTGAAGATGAAGATATCCAGATTGGTCCCGTGGATGATCCTGGGGTCTCTGCTATATGTATCTATCATTTGTGTTTTCCATAGCAAATATTCAGCGTTTTTTGTTCCACGCAGCCTCATGAAATTTTTCTCCCAAAATGCCACAATTCAAATAGAAGATATACCTGCTGCACAGATTTTCTCTTTTGCTGCTGAGCTCTTAGTGCCACTGCTTATTTTCCTTGTTGCTGTTCTGCTCTTGATTTTTTCTTTGGGGAGGCACACCTGGCAAATGAGAAACACAGTAGCCAGCAGCAGGGTACCTGGCAGGAGTGGCCACATCAGCGCATGGCTGTCCATCCTGTCCTTCCTAATCCTCTATGTTTCCCACTACATGATGAAAGCTTTTCTCTGTTCTATAAGGTTTCACATCAGAAGGTTCACCTTTATGCTCTGCATCCTTGTGATTGGCACATACCCTTGTGGACACTCTCTCATCTTAATTTTAGGAAATCCTAAATTGAAACAAAATGCAAAAAAGTTCCTCCTCCACAGTAAGTGCTGCCAGTAA

>Aotus nancymaae

ATGCTGGAGTCTGACCTCATTACCTGCTTTCTTCTTTCAGTCATACAATTTCTTCTTGGGATTTTCATGAATGGCATCATTGTGGTGGTGAATGGCATTGACTTGATCAAGCACAGAAAAATGGCTCCAGTGGATCTCCTTCTTTCCTGCCTAGCACTTTCCAGAACTTTTCTGCAGTTGTTCATCTTCTACATTAATCTGGTTGTTCTCTTCTTGATGAAATTAATCATGTATCCTGAGAGTTTTATAATTATCATGTTTATAAACGAATTGGAACTTTGGCTTGCCACATGGCTTGGTGTTTTCTACTGCACCAAGGTTTCCAGCGTCCCTCACCCACTCTTCATCTGGTTGAAGATGAAGATATCCAAGTTGGTCCCATGGATGATCCTGGGGTCTCTGCTGTATGTATCTATCATTTGTGTTTTCCGTACCAAATATTCAGTGTTTTTTGTTCCACACAGCCTCCTGAACTTTTTCTCCCAAAATGCCACAGTTCAAATAGAAGATATACCTGCTATACAGATTTCCTTTTTTGCCACTGAGCTCTTAGTGCCACTGCTTATTTTCCTTCTTGCTCTTCTGCTCTTGATTTTTTCTTTGGGGAGGCACGCCTGGCAAATGAGAAACACAGTAGCCAGCAGCAGGGTTCCTGGCAGGAGTGGCCACATCAGCGCATGGCTGTCCATCCTGTCCTTCCTAATCCTCTATGTTTCCCACTACATGATGAAAGCTTTTCTCTATTGTATACGGTTTCACATCAGAAGGTTCATCTTTATGCTCTGCATCCTTGTGATTGGCACATACCCTTCTGGACATTCTCTCATCTTATTTTTAGGAAATCCTAAATTGAAACAAAATGCAAAAAAGTTCCTCCTCCACAGTAAGTGCTGTCAGTGA

>Tarsius syrichta

ATGCTTGAGTTTCACCTCATCGTCTATTTTCTTTTTGTGCTGATCCAGTTTCTTGTTGGGGTTTTTGCAAATGGCTTCATTGTGGTGGTGAACACCATTGACTTGATCAAACAGAGAAAATTGGCTCCATTGAACCTACTTCTTTTCTGCCTGGCGATTACTAGACTCTGTCTGCAATTGCTCATCTTCAACATTAATCTGGTTATTCTCTCCATGATAGAATGGCTTATATTTCCTTCAAATTTTTCAATTCACATGCTTGTAAATGAATCGGAACTTTGGTTTGCCACATGGCTGGGTGTTTTCTACTGCGCCAAGATTGCCACTGTCCCTCACCCACTCTTCATTTGGTTGAAGATGAGGATAACCAAGTTGGTGCCGTGGCAGATTCTTGGGACTCTGTTGTATACATCTATCATTTGTATTTTCCAAAACAATTACACGTGGCGTATTCCTCCAGCATTCTCAGTGCACCTTTTCTCCCCAAACGCGACAACTCAAATCACAGAAGTAACTGCCTTGCAGCTTGCCTTTCTCGTCTTTGGGTTCTCAACGCCCTTACTGATTTTCTTGGTTGCAGTTCTGCTCTTGATTTTTTCCCTGGGAAAACACTCCCGGCAGATGAGAAGCACAACGATGGGCAGCAGGGATTCTCGCAGGAGTGCCCACATCAGTGCGATGCTATCCATCTTTTCCTTCCTAGTCCTGTACTTTTCTCACTATATGATGGCAGCCTTACTGTCTTCTCAATATTTCCAGCTCAGAAGCTTTGTCTTTCTCTTCTGCATGTTGGTGGTTGGTACATACCCCTCTGGACACTCTCTCATCTTAATTTTAGGAAATCCTAGACTGAAACAAAGTGCAAGGAAGTTTCTCTTCTACAGTAAGAGCTGTCAGTAA

>Otolemur garnettii

ATGCTAAACTCTCCCCTCATTATCTCTTTTCTTTTTATGCTGACGCAATTCCTTTTTGGGATTCTTGCAAATAGCATCATAGTGGTGGTGAATGGCATTGGGCTGGTCAAGTGGAAGAGAATGCCTCCCCTGGATCTCCTTCTTTCTTGCCTGGCGCTTTCTAGAATTTGTCTGCAGGGAACCATCTTGCATTTTTGTCTAACTGTACTATCTTTGACAGAGGTTTTTAGGTTTTCTCGGAATTTTGTGGCCTACCTGTTTGTAAGTGAATCAGAACTCTGGCTTGCCACGTGGCTGGGTGTTTTCTACTGTGCCAAGATCGCCAACATTTCTCATCCTTTCTTCTTCTGGTTGAAGATGAGGATAACCAAGCTGGTGCCATGGCTGATCCTGGGCTCTCTGTTCTATGCATCTGCCACTTGTGTTTTGCACAGCAACTACCAATGGATTATTGCCCAAAACTTCTCAGTGACCTTTGTCTTCAAAAATACAGTAGCTCAAATGAAAGAAAAATTCGCTTTACAGTTTTTCTTGCTGATTGTTGAGGTCTCCATACCGTTATTTATCTTCCTTGCTGCGGGTCTCCTGCTGGTTTTTTCCCTGGGGAGACACACCCAGCAGATGAGAAACAGGGCTGCAGGCGCCGGGGACACTCACAGGTATGCCAGCGTCCAGGCGATGCTGTCCATCCTGTCCTTCCTCATCCTCTACTTCTCCCACTTCATAGCGATTGCCTTGCTCACTCTTGAAAATTTTCAGTATGGAAGCTTAAATATTCTGTTCTTCATCCTGATGTTTGGTACCTACCCCTCTGCACACTCTATCATCTTAATTTTAGGAAATCCTAAACTAAAACAAAATGCAAAGAAGTTCTTCCTTCACAGTAAGTGCTGTCAGTGA

>Daubentonia madagascariensis

ATGCTAGAGTCTCACCTCATTATATGTTTTCTTTTTGCAGTGATACAATTTCTTATTGGGGTTTTTGCAAATAGCATCATCGTGGTGGTGAATGGCATTGGCTTGATCAAGCAGAGAAAAATGGCTCCGCTGGATCTCCTCCTTTCCTGCCTGGCAGTTTCTAGAATTTGCCTGCAGTTGCTCATCTTCTACACTCATCTGGGTGTTCTCTCATTGGTAGAATTCCTTACATTTTCTGAGAGTTTTACAATCAGCTTTCTTACAAGTGAATCGGAACTTTGGTTCGCCACGTGGCTGGGCGTTTTCTACTGCGCCAAGATTGCCAACGTTTCTCACCCGCTCTTCTTCTGGCTGAAGATGAGGATATCCAAGCTGGTGCCGTGGTTGATCCTGGGGTCTCTGCTATATGTATCTACTGCTTGTGTTTTCCACAGCAAATATATACAGCCTATTCCCCAAAACCTCTCTGTGGGCTTTTCCTCTAAAAATATAATGACTCAAATCAAAGTTAAGTTTGCTTTGCAGTATTTCTTTTTCGTTATGGGGTTCCCTGTGCCATTACTTATCTTCCTTGTTGCTGTTCTGCTCCTGGTCTTTTCCCTGGGGAGACATGCACAGCAGATGAGAAACATGGCGGCAGGAAGCAGGGACCCTCGCAGGAGTGCCGACGTCCACGCGATGCTGTCCATCCTGTCCTTCCTAATCCTCTACTTCTCCCACTACACGATGGAAGGGTTGTTCTCGTCTCAAAAATTTCAGCTCGGAAGCTTCAGCTTTCTGTTCGGCATTTTGGTGCTTGGTACATACCCCTCTGTACACTCTGTCATCTTAATTTTAGGAAATCCTAAACTAAAACAAAATGTAAAGAAGTTCCTCCTTCACAATAAGTGCTGTCAGTGA

>Propithecus coquereli

ATGCTAGAGTCTCACCTCATTATCTCTTTTCTTTTTGTGGTGATACAATTTCTTATCGGGATTTTTGCAAATAGCATCATCGTGGTGGTGAATGGCATTGGCGTGATCAAGCAGAGAAAAATGACGCCACTGGGCCTCCTTCTTTCCTGCCTGGCAGTTTCTAGAATTTTTATGCAGTTGTTCATCTTTTTAAATCACATGGATGCTCTCTCCTTGGTAGAAATCCTCATACCTTCTGACGGTTTTGGAATCAGCTCGTTCATAAATCAATCGGAACTTTGGTTTGCCACATGGCTGGGTGTTTTCTACTGTGCCAAGATTGCCAACATTTCTCACCCACTCTTCTTCTGGCTGAAGATGAGGATATCCAAGCTGGTGCCATGGCTGATCCTGGGGTCTCTGCTATATGCATCTACCTCTTGGGTTTTCCACAGCAAATATATATGGCCTATTTCCCAAAACCTCTCAGTAGGCTTTTCCTCCGAAAATATGAGGACTCAAAAGAAAGAAAGTCTTGCTTTGGATTATTTCTTTCTTGTCCTGGGGTTCCCCATTCCATTATTTATCTTCCTTGTTGCTACTCTACTCCTGGTTTTTTCCTTGGGGAGACACACACGGCAGATGAGAAACATGACGGCCGGCACCAGGGACCCTCGCATGAGTGCCCACATCCATGCCGTGCTCTCCATCCTGTCCTTCCTAATCCTCTTCTGCTTCCACTACACGATGGAATTGTTGATCTCTTTTCAAACTCTTCAGTTCAGAAGACTCAACTTTCTGTTCGTCATGTCTGTACTTGGTATGTACCCCTCTATACACTCTACCATCTTAATTTTAGGAAATCCTAAACTAAAACAAAATGCAAAGAAGTTCCTCCTCCACAATAAGTGCTGTCTGTGA

>Microcebus murinus

ATGCTAGAGTCTCACCTTATTATCTATTTTATTTTGGGTGTGATACAGTTTCTTATTGGGATTTTTGCAAATAGCATCATCGTGGCGGTGAATGGCATTGGCGTGATCAAGCAGAGAAAAACAGCTCCACTGAATCTCCTCCTTTCCTGCCTGGCAGTTTCTAGAATTTTTCTACAGTTGTTCATTTTTTTCACTCATCTGGCTGTTCTCTCCTTGATAGAATTCCTTACACTTTCTGAAGGCTTTGGAATCAGCCTGTTCATAAATGAATCGGAACTTTGGTTTGCCACATGGCTGGGTGTTTTCTACTGCACCAAGATTGCCAACATTTCTCACCCGCTCTTCTTGTGGTTGAAGATGAGGATATCCAAGCTGGTGCCGTGGCTGATCCTGGGGTCTCTGCTATGTGCATCTACCTCTTGTGTTTTCCACAGCGAATATATGTGGCCTATTCCCCAAAACCTCTCCATGGGCTTTACCTCCAAAAACACGAGGACTCAAATGAAAGAAACTCTTGCTTTTCAGTATTTCTTTTTTGTTCTGGGGTTCCCTATGCCACTATTTATCTTCCTTGTTGCTATTCTACTCCTGGTTTTTTCCTTGGGGAGACACACATGGCAGATGAGAAATATGGCGGCAGGCACCAGGGACCCCCGCGTGAGTGCCCACATCCATGCCATGCTCTCCATCCTGTCCTTCCTAATCCTCTTCTTCTTCCATTACATAATGGAAATATTGCTCTCTTCTCAAACTCTTCAGCTCAGAAGCTTCAACTTTCTATTCTGCATGTCTGTGCTTGGTACATACCCTTCTGTACACTCTACCATCTTAATTTTGGGAAATCCTAAACTAAAACAAAACGCAAAGAAGTTCCTCCTCCACATTAAGTGCTGTCAGTGA

>Prolemur simus

ATGCTAGAGTCTCACTTCATTATCTATTTTCTTTTTGTGGTGATACAATTTCTTACCGGGATTTTTGCAAATAGCATCATCGTGGTGGCGAATGGCATAGGCATGATCAAACAGAGAAAAATGGCTCCACTGGGTCTCCTTCTTTCATGCCTGGCAGTTTCCAGAATTGGTCTGCAGGTGTTCATCTTTTTCACTCATCTGGCTATTCTCTCCGTGATAGAATTTCTTACATTTTCTAACGGTTTTGGAATCAGCTTGTTCATAAATGAATCGGAACTTTGGTTTGCCACATGGCTGAGTGTTTTCTACTGTGCCAAGATTGCCAACATTTCTCACCCGCTCTTCTTCTGGTTGAAGATGAGGATATCCAAGCTGGTGCCATGGCTGATCCTGGGGTCTCTACTATATTCGTGTACCTCTTGTGTTTTGCACAGCAAATATATATGGCTTATTCCCCAAAACCTCTCGGTGGGCTTTTCCTCCAAAAACATGAAAGAAAGTCCTGCTTTACGGTATTTCTTTTTTGTCCTGGGGTTCCCCATGCCATTATTTATCTTCCTTGTTGCTGTTCTACTCCTGGTTTTTTCCCTGGGGAGACACACACGGCAGATGAGAAACATGGCGGCAGGCACCAGGGACCCTCGCAGGAGCGCCCACATCCATGCCATGCTCTCCATCCTGTCCTTCCTAATCCTCTTCTTCGTCCACTATACAATGGAAATGTTGATCTCTTCTCAAACTCTTCAGTTCAGAAGCTTCAGCTTTCTATTCTGCATGTCTGCACTTGGTACATACCCCTCTGTACACTCTACCATCTTAATTTTAGGAAATCCTAAACTAAAACAAAATGCTAAGCAATTCCTCCGCCACTTTAAATTATGTCAGTGA

>Eulemur flavifrons

ATGCTAGAGTATCACCTCATGATCTATTTTATTTTTGTGGTGATACAATTTCTTACTGGGATTTTTGCAAATAGCATCATCGTGGTGGTGAATGGCATTGGCGTGATCAAGCAGAGAAAAATGGCTCCACTGGGTCTCCTTCTTTCCTGCCTGGCAGTTTCCAGAATTGGTCTGCAGTTGTTCATCTTTTTCACTCATCTGGCTATTCTCTCCATGATAGAATTTCTTACACTTTCTAATGGTTTTGGAATCAGCTTGTTCATAAATGAATCGGAACTTTGGTTTGCCACATGGCTGGGTGTTTTCTACTGCGCCAAGATTGCCAACATTTCTCACCCACTCTTCTTCTGGTTGAAGATGAGGATATCCAAGCTGGTGCCATGGCTGATCCTGGGGTCTCTGCTATATTCGTGTACCTCTTGTGTTTTGCACAGCAAATATATATGGCTTATTCCCCAAAACCTCTCGGTGGGCTTTTCCTCCAAAAACATGAAAGAAAGTCTTGCTTTGCGGTATTTCTTTTTTGTGCTGGGGTTCCCCACGCCATTATTTATCTTCCTTGTTGCTATTCTACTCCTGGTTTTTTCCCTGGGGAGACACACACGGCAGATGAGAAACATGGCGACAGGCACCAGTGACCCTCGCAGGAGTGCCCACATCCATGCCATGCTCTCCATCCTGTCCTTCCTAATCCTCTTCTTCGTCCACTATACAATGGAAATGTTGATCTCTTCTCAAACTCTTCAGTTCAGAAGCTTCAGCTTTCTATTCTGCGTGTCTGTACTTGGTACATACCCCTCTGTACACTCTACCATCTTAATTTTAGGAAATCCTAAACTAAAACAAAATGCAAAGAAGTTCTTCCGCCACATTAAATTATGTCAGTGA

>Eulemur macaco

ATGCTAGAGTATCACCTCATTATCTATTTTATTTTTGTGGTGATACAATTTCTTACTGGGATTTTTGCAAATAGCATCATCGTGGTGGTGAATGGCATTGGCGTGATCAAGCAGAGAAAAATGGCTCCACTGGGTCTCCTTCTTTCCTGCCTGGCAGTTTCCAGAATTGGCCTGCAGTTGTTCATCTTTTTCACTCATCTGGCTATTCTCTCCATGATAGAATTTCTTACACTTTCTAATGGTTTTGGAATCAGCTTGTTCATAAATGAATCGGAACTTTGGTTTGCCACATGGCTGGGTGTTTTCTACTGTGCCAAGATTGCCAACATTTCTCACCCACTCTTCTTCTGGTTGAAGATGAGGATATCCAAGCTGGTGCCATGGCTGATCCTGGGGTCTCTGCTATATTCGTGTACCTCTTGTGTTTTGCACAGCAAATATATATGGCTTATTCCCCAAAACCTCTCGGTGGGCTTTTCCTCCAAAAACATGAAAGAAAGTCTTGCTTTGCGGTATTTCTTTTTTGTGCTGGGGTTCCCCACGCCATTATTTATCTTCCTTGTTGCTATTCTACTCCTGGTTTTTTCCCTGGGGAGACACACACGGCAGATGAGAAACATGGCGGCAGGCACCAGTGACCCTCGCAGGAGTGCCCACATCCATGCCATGCTCTCCATCCTGTCCTTCCTAATCCTCTTCTTCGTCCACTATACAATGGAAATGTTGATCTCTTCTCAAACTCTTCAGTTCAGAAGCTTCAGCTTTCTATTCTGCGTGTCTGTACTTGGTACATACCCCTCTGTACACTCTACCATCTTAATTTTAGGAAATCCTAAACTAAAACAAAATGCAAAGAAGTTCTTCCGCCACATTAAATTATGTCAGTGA
